# Bone Marrow-Derived Mesenchymal Cell Differentiation toward Myogenic Lineages: Facts and Perspectives

**DOI:** 10.1155/2014/762695

**Published:** 2014-06-26

**Authors:** Daniela Galli, Marco Vitale, Mauro Vaccarezza

**Affiliations:** ^1^Department of Biomedical, Biotechnological and Translational Sciences (S.Bi.Bi.T.), University of Parma, Via Gramsci 14, 43126 Parma, Italy; ^2^Department of Human, Social and Health Sciences, University of Cassino and Southern Lazio, Via Sant'Angelo, Località Folcara snc, 03043 Cassino, Italy; ^3^School of Biomedical Sciences, University of Queensland, Brisbane, QLD 4072, Australia

## Abstract

Bone marrow-derived mesenchymal stem cells (BM-MSCs) are valuable platforms for new therapies based on regenerative medicine. BM-MSCs era is coming of age since the potential of these cells is increasingly demonstrated. In fact, these cells give origin to osteoblasts, chondroblasts, and adipocyte precursors *in vitro*, and they can also differentiate versus other mesodermal cell types like skeletal muscle precursors and cardiomyocytes. In our short review, we focus on the more recent manipulations of BM-MSCs toward skeletal and heart muscle differentiation, a growing field of obvious relevance considering the toll of muscle disease (i.e., muscular dystrophies), the heavier toll of heart disease in developed countries, and the still not completely understood mechanisms of muscle differentiation and repair.

## 1. Introduction

Regenerative medicine and its exceptional potential in clinics [1–4] are based on the discovery of the properties of stem cells. Till et al. [5] showed that single cells could yield multilineage descendants while preserving the multipotency of the mother cell. The researchers gave substance to the idea of a stem cell and gave us methods to define the cardinal properties of those cells—self-renewal and differentiation [5].

This latter discovery paved the way to subsequent explosion of the stem cell biology, one of the most fast developing and interesting areas of biomedical research, and the possibility to manipulate human stem cells to obtain new cells with a needed phenotype was exploited since the discovery of these cells.

In the beginning, only the hematopoietic derived lineage was pursued: more knowledge of the biology of these cells and their plasticity allowed for larger manipulation with the possibility to obtain several cell lineages with amazing perspectives on a clinical point of view [1–4].

Remarkably, bone marrow contains different types of progenitors: the hematopoietic progenitors that give rise to all the hematopoietic cell types (haematopoietic stem cells: HSCs); the mesenchymal stem cells (BM-MSCs) that are able to differentiate into chondroblasts, osteoblasts, and adipocyte precursors and the endothelial progenitors that give rise to the inner layer of vessels.

These cells communicate through cell contacts, growth factors, cytokines, and extracellular matrix proteins, creating microdomains or niches and regulating their self-renewal, differentiation, and quiescence [6]. Recent evidence uncovered an unprecedented partnership between the two distinct somatic stem cell types that is indicative of a unique niche in the bone marrow made of heterotypic stem cell pairs [7, 8].

In the last 15 years, the induction of both hematopoietic and mesenchymal cells to different mesodermal fates like cardiomyocytes and skeletal myoblasts* in vitro*,* ex vivo*, and* in vivo *has been reported. Indeed, this will be the focus of this short review: the possibility to derive muscle cells from several cell precursors is of pivotal interest not only to better understand muscle physiology and metabolism, but also to define a road map for the regeneration of diseased muscle (even heart muscle) that often is not able to repair itself to a complete “restitutio ad integrum.”

Initially, mouse hematopoietic progenitors have been identified as Lineage (Lin)−, c-Kit+, and Sca1+ cells [9], but later it emerged that almost all progenitors were characterized by the absence of CD34 [10]. In humans, it is the contrary, as the first marker of HSCs that was discovered was CD34 [11]; hematopoietic precursors also showed positive to different isoform of CD45 [12]. HSCs marker physiology and discussion are beyond the scope of this review: for a complete report and comparison on mouse and human hematopoiesis we suggest recent papers written by Doulatov et al. [13]. The importance of the bone marrow niche and the relationship in the microenvironment between HSCs, BM-MSC, and also endothelial precursors cells (EPC) are highlighted by the studies by Scadden [6, 8].

### 1.1. Skeletal Muscle Differentiation of HSCs

An active role of hematopoietic cells to muscle regeneration has been established [14], but the related molecular mechanisms are still unclear.

*In vitro* study performed by Polesskaya et al. in 2003 [15] demonstrated that CD45+/Sca1+ cells isolated from muscle can form myogenic clones when cocultured with skeletal myoblasts. Just one year later, Sherwood et al. [16] demonstrated that CD45+/Sca1− cells, derived from muscle, showed* in vivo* myofiber-forming ability but were not able to differentiate into myocytes either alone or in coculture* in vitro*.

Hence, attempts were made to characterize mechanisms of* in vivo* contribution of bone marrow-derived cells to myofibers and it emerged that hematopoietic stem cells participated in muscle regeneration by direct fusion without specification of a myogenic program [17, 18].

First approaches on muscle disease therapy did not have positive outcomes. In fact* in vivo* transplantation of hematopoietic fraction isolated from bone marrow did not restore dystrophin expression in dystrophic dogs [19].

Recently, Xynos et al. [20] demonstrated that CD45+/Sca1+ cells both isolated from bone marrow and muscle did not express key myogenic factors like Pax7 and MyoD, although they underwent myogenic reprogramming and participated in myofiber fusion. These results suggest that CD45+ cells isolated from muscle form a population that contributes to tissue regeneration that is distinct from satellite cells.

### 1.2. Cardiac Muscle Differentiation of HSCs

Studies performed in 2001 in mice by Anversa's group [21] proved efficient regeneration of myocardium after ischemia by transplantation of Lin− cKit+ HSCs. Later they also showed that stem cell factor (SCF) and granulocyte colony-stimulating factor (GCSF) mobilization of Lin−, c-Kit+ hematopoietic stem cells significantly decreased infarct size, cavitary dilation, and diastolic stress [22].

In this paper, they demonstrated cardiac differentiation of transplanted HSCs; but this result was not confirmed by other groups [23, 24]. Instead, it emerged that HSCs generate cardiomyocytes with low frequency by fusion with resident cells [25, 26] but not by active cardiac differentiation.

Emerging roles in cardiac disease therapies have been demonstrated for hematopoietic cytokines like GCSF, granulocyte macrophage colony-stimulating factor (GM-CSF), SCF, Flt-3 ligand, and erythropoietin (EPO). In fact, these molecules induce mobilization and homing of HSCs and also exert cytoprotective effects like reduction of apoptosis and induction of angiogenesis [27].

Currently, phase I and II clinical trials (REPAIR-ACS, REGEN-AMI, TIME, and LATE TIME) are ongoing with bone marrow-derived HSCs in the therapy of myocardium infarct [28]. In TOPCARE clinical trial of heart failure affected patients, BMSCs cell transplantation was associated with a significant, though moderate, amelioration of left ventricular ejection fraction. Instead, isolated bone marrow HSCs were not as beneficial (AMI clinical trial) suggesting that all the bone marrow stem cell populations are important for cardiac recovery. There is an ongoing hot debate on the conflicting results of resident cardiac stem cells and/or migrating blood derived stem cells in heart regeneration and repair: several publications question the appropriateness of the animal models as well as the tracking systems utilized to identify cells involved in regeneration and repair [29–35].

## 2. Bone Marrow-Derived Mesenchymal Stem Cells (BM-MSCs)

The nonhematopoietic bone marrow-derived cells can be cultured as plastic adherent cells and are defined by different names: bone marrow stromal cells, bone marrow mesenchymal stem cells [36]. Of note, these cells defined as MSCs are so labeled because of their function* in vitro*, not* in vivo*, and we have to consider that mesenchymal cells grown* in vitro* have extensive biological testing but lack rigorous confirmation that they reflect an* in vivo* stem cells population. In this minireview, they will be called bone marrow-derived mesenchymal stem cells (BM-MSCs). Initially described by Friedenstein et al. [37, 38], BM-MSCs were defined by Caplan [39] who considered their differentiation ability towards other mesenchymal lineages beside the osteogenic one.

BM-MSCs are characterized by the expression of CD29, CD73, CD105, CD90, CD44, and CD146 surface markers, all of them reviewed in [40, 41]. However, all these markers are expressed in other bone marrow cells, thus creating an issue for prospective isolation. Recently, other markers such as CD271 and W8-B2/MSCA-1 have been suggested for BM-MSCs purification but definitive confirmations by other laboratories are still lacking [42].

Basically, BM-MSCs are defined as plastic adherent cells that express CD105, CD90, and CD73 but lack the expression of pan-leukocyte, endothelial or primitive haematopoietic, and monocytic or B cell markers and lack HLA class II antigens on the cell surface [43]. Usually, bulk cell populations must demonstrate trilineage differentiation into osteoblasts, adipocytes, and chondroblasts [43].

Bianco's group [44] showed that human CD146+CD45-expression marked self-renewing osteoprogenitor cells containing all the BM CFU-activity and capable of generating a heterotopic BM niche in a subcutaneous transplantation model. More recent studies have suggested that a similar frequency of CFU-Fs could be recovered from CD271+CD146low/CD45-human BM cells [45]. Another independent study has revealed that the intermediate filament protein Nestin marked perivascular stromal cells (Nestin+CD31-CD45-) that contain all the CFU-F activity within the BM and the exclusive capacity to form clonal spheres (termed mesenspheres) when cultured in nonadherent conditions [46]. Because only a fraction of CFU-Fs represents truly BM-MSCs, more work is required to define BM-MSCs and distinguish them from differentiated progeny.

BM-MSCs represent the optimal candidate for cell therapy because they can be easily obtained from a bone marrow aspirate and expanded on a large scale before autotransplantation, avoiding ethical problems. Recently, it emerged that the principal role of BM-MSCs is the protection of the host tissue after transplantation: they produce different cytokines that reduce apoptosis and induce neovascularization in neuronal and cardiac tissues [47, 48].

### 2.1. Skeletal Muscle Differentiation of BM-MSCs

In addition to the classical trilineage potential, BM-MSC may also differentiate into other mesodermal or even nonmesodermal cell types, such as myoblasts, hepatocytes, and neural cells [34, 49, 50].* In vitro* skeletal myogenic induction of BM-MSCs was demonstrated by Dezawa et al. in 2005 [51]. Cells were treated with basic fibroblast growth factor (bFGF), platelet derived growth factor-AA, and neuregulin. After 3 days the cells were transfected with Notch1 intracellular domain (NICD) containing plasmid. Myotube formation and contraction together with expression of late markers such as myogenin and myosin heavy chain were observed after treatment with supernatant of the original BM-MSCs.

Muscle regeneration has been tested after transplantation of BM-MSCs obtained from GFP-transgenic mice [52]. Immunosuppressed mdx-dystrophic mice received GFP-positive BM-MSCs after damage induced with cardiotoxin treatment. After 4 weeks, histological analysis showed a significant number of GFP+/dystrophin+ fibers. At that point, if the same muscles were treated with cardiotoxin, it was possible to observe GFP-positive immature myofibers after 2 weeks. This important result showed that transplanted BM-MSCs contained a subpopulation that acts as satellite cells, retaining capability for future muscle regeneration.

Similar experiments were performed by de la Garza-Rodea et al. [53]. In this case BM-MSCs were transduced with a LacZ-coding lentivirus. Four weeks after cell transplantation, lacZ positive myofibers corresponded to 5% of total myofibers, equally distributed along all the muscle, thus confirming myogenic differentiation of BM-MSCs, though with low frequency.

### 2.2. Cardiac Muscle Differentiation of BM-MSCs

*In vitro* cardiac differentiation of BM-MSCs after addition of the demethylating agent 5-azacytidine was showed by different groups [54–56]. Typically, 2–4 weeks after treatment, BM-MSCs changed morphology and expressed cardiac specific genes (e.g., Nkx2.5, Gata4, and Mef2C). Besides 5-azacytidine treatment, other molecules have been used to induce cardiac differentiation of BM-MSCs: dexamethasone and ascorbic acid [57]; bone morphogenetic protein-2 (BMP-2); and fibroblast growth factor-4 (FGF-4) [58]. The coculture with cardiomyocytes has also been used to induce cardiac differentiation of BM-MSCs [59–61]. Positive effects on cardiac differentiation of BM-MSCs, when put in coculture with cardiomyocytes, suggest a role of cell-cell communication for the induction of* in vitro* differentiation.

There is an open controversy on the role of BM-MSCs in cardiac therapy. There are different papers that show engraftment of BM-MSCs in models of myocardium infarct with functional amelioration. Toma et al. [62] demonstrated that 0.44% of injected human BM-MSCs engrafted immunodeficient murine hearts and adopted mature cardiac phenotype 2 weeks after injection.

In a model of chronic cardiomyopathy, Quevedo et al. [63] showed that 10% of the injected cells differentiated into new vessels, while the remaining 76% was found in the interstitial cardiac compartment without evidence of lineage commitment.

In contrast, two groups did not find either engraftment or cardiac differentiation of BM-MSCs in a sheep model of myocardium infarct [64] or dog model of ischemic cardiomyopathy [65], suggesting that other experiments are still necessary to clarify the role of BM-MSCs in cardiac repair.

Moreover, functional recovery could not be explained simply by differentiation of injected BM-MSCs, since the number of differentiated cells corresponds to 10–15% of the total. Therefore, other possible mechanisms of action have been investigated. In particular, since BM-MSCs secrete different antiapoptotic and angiogenic factors (e.g., bFGF, HGF, IGF1, and VEGF), different groups have studied whether or not these molecules could mediate left ventricular functional improvement and reduction of infarct scar [66–69]. Although a positive effect of BM-MSCs conditioned media administration was encountered, it was not the same as obtained by BM-MSCs injection. Of note, paracrine factors do not recruit cardiac stem cells (CSCs) in the infarct area as BM-MSCs do [70]. This result obtained in a porcine model was not confirmed in mice, suggesting that more experiments should be performed to understand the role of BM-MSCs in myocardium infarct recovery [71].

### 2.3. Smooth Muscle Differentiation of BM-MSCs

Direct smooth muscle differentiation of BM-MSCs was investigated* in vitro* by comparing expression of smooth muscle markers like alpha-smooth muscle actin (ASMA) and h1-calponin (CALP) in BM-MSCs cultured on extracellular matrix (ECM) proteins: laminin (LM), collagen type IV (Col-IV) and fibronectin, or normal plastic. Results showed increased expression of ASMA and CALP* in vitro* [72].

TGFbeta-1 has been shown to be important for smooth muscle differentiation of BM-MSCs, although, in this case, cell-cell contact is required for resembling tissue development* in vivo* [73]. On the contrary,* in vivo* angiogenesis had been shown with both mesenchymal precursors and osteoblasts in transplanted scaffolds, but it was strongly related to VEGF production [74]. This suggests that new angiogenesis is independent by vascular differentiation of BM-MSCs.

## 3. Conclusions

Bone marrow stem cells represent a useful source of progenitors for cell therapy thanks to the fact that they can be isolated by a relatively simple method which is a single marrow biopsy. HSCs efficiently reconstitute blood cells, but they have a positive effect on muscle disease therapy. BM-MSCs cells normally differentiate towards osteocytes, chondrocytes, and adipocytes. Like HSCs, BM-MSCs present plasticity* in vitro* towards other mesodermal cell types such as cardiac and skeletal myocytes. However, for both HSCs and BM-MSCs, it is now widely recognized that they participate in cell therapy also producing cytokines and growth factors that have antiapoptotic and angiogenic effect. Our understanding of what constitutes an HSC and BM-MSC, its metabolic activities, and therapeutic potential has improved considerably since the initial isolation of colony-forming cells a few decades ago. The benefits of heterogeneous cell populations (including hematopoietic stem cells, endothelial progenitor cells, and platelets) and limitations of allogeneic BM-MSCs require further basic and clinical investigation. On the basis of the preliminary reports of safety and efficacy in several medical specialties, autologous cell therapies (freshly harvested or culture-expanded cells) represent a method to treat conditions that currently are inadequately treated and generally result in poor outcomes or invasive surgery [75, 76].

More clinical data is necessary to determine the* in vivo* distribution and therapeutic mechanisms of HSCs and in particular of BM-MSCs to optimize their use in a personalized regenerative medicine portfolio. This process will require the collaborative efforts of physicians, scientists, and industry and regulatory agencies to translate nature's basic regenerative element into the continuum of clinical care.
